# Iterative Reconstruction with Dynamic ElasticNet Regularization for Nuclear Medicine Imaging

**DOI:** 10.3390/jimaging11070213

**Published:** 2025-06-27

**Authors:** Ryosuke Kasai, Hideki Otsuka

**Affiliations:** Department of Medical Imaging/Nuclear Medicine, Institute of Biomedical Sciences, Tokushima University, 3-18-15 Kuramoto, Tokushima 770-8509, Japan; kasai-r@tokushima-u.ac.jp

**Keywords:** image reconstruction, ElasticNet, regularization, tomography

## Abstract

This study proposes a novel image reconstruction algorithm for nuclear medicine imaging based on the maximum likelihood expectation maximization (MLEM) framework with dynamic ElasticNet regularization. Whereas conventional the L1 and L2 regularization methods involve trade-offs between noise suppression and structural preservation, ElasticNet combines their strengths. Our method further introduces a dynamic weighting scheme that adaptively adjusts the balance between the L1 and L2 terms over iterations while ensuring nonnegativity when using a sufficiently small regularization parameter. We evaluated the proposed algorithm using numerical phantoms (Shepp–Logan and digitized Hoffman) under various noise conditions. Quantitative results based on the peak signal-to-noise ratio and multi-scale structural similarity index measure demonstrated that the proposed dynamic ElasticNet regularized MLEM consistently outperformed not only standard MLEM and L1/L2 regularized MLEM but also the fixed-weight ElasticNet variant. Clinical single-photon emission computed tomography brain image experiments further confirmed improved noise suppression and clearer depiction of fine structures. These findings suggest that our proposed method offers a robust and accurate solution for tomographic image reconstruction in nuclear medicine imaging.

## 1. Introduction

Image reconstruction is a fundamental component of nuclear medicine imaging, enabling the visualization of physiological and biochemical processes within the human body [1,2]. Modalities such as positron emission tomography and single-photon emission computed tomography (SPECT) rely on the detection of gamma rays that are emitted from radiotracers administered to the patient [3,4]. The reconstruction process aims to estimate the spatial distribution of these tracers from projection data that are acquired at multiple angles using gamma cameras or detectors [5].

Mathematically, this task is formulated as an inverse problem, the goal of which is to recover an image from its line integrals (i.e., the sinogram) [6]. However, this problem is typically ill-posed and underdetermined, particularly in clinical settings involving limited acquisition angles, low-dose imaging, or short acquisition times [7,8]. These conditions yield incomplete and noisy data, making the inversion process highly sensitive to noise and measurement errors. Various regularization techniques have been employed to constrain the solution space and improve the stability and quality of the reconstructed images to address these issues [9].

Conventional emission tomography approaches can be broadly categorized into analytical methods, such as filtered backprojection (FBP) [10,11], and iterative statistical methods, such as maximum likelihood expectation maximization (MLEM) [12]. Although FBP is computationally efficient and widely used, it is prone to artifacts and not highly robust to noise, especially under low-dose conditions [13]. In contrast, the MLEM algorithm models the projection data as Poisson-distributed, in accordance with the nature of radioactive decay. This probabilistic framework yields improved reconstructions, particularly under noisy or sparse data conditions. However, a common drawback of MLEM is that noise tends to accumulate with the iterations, degrading the image quality and obscuring the fine structural details [14,15,16,17,18].

Regularization terms have been incorporated into the MLEM framework [19,20] to mitigate this problem. Among the regularization terms, the L1 and L2 norms are the most commonly used. L1 regularization promotes sparsity and preserves edges, making it effective for images with sparse representations, such as those in the wavelet or gradient domain [21,22,23]. However, it can introduce undesirable artifacts such as piecewise constant regions or blocky textures. In contrast, L2 regularization enhances noise suppression across the image but may smooth out sharp boundaries and remove clinically important high-frequency details [24,25].

To combine the strengths of both the L1 and L2 regularization, ElasticNet regularization has been proposed, which introduces a convex combination of both norms [26]. Originally developed for variable selection and shrinkage in statistical learning, ElasticNet has recently gained attention in image processing tasks, including image reconstruction [27,28]. In these contexts, ElasticNet has been shown to preserve structural details while maintaining effective noise control.

Despite its potential, the use of ElasticNet in emission tomography remains limited. Furthermore, prior approaches typically employ static weighting between the L1 and L2 terms throughout the reconstruction process, which may not be optimal as the reconstruction evolves. In early iterations, strong L2 regularization may help stabilize the image, while later iterations may benefit from L1-driven sparsity to sharpen details.

To address this, we propose a novel image reconstruction algorithm based on the MLEM framework with dynamically weighted ElasticNet regularization, in which the relative contributions of L1 and L2 terms are adaptively modulated during the iterative process. By adapting the regularization profile based on iteration count or image characteristics, the method allows for greater flexibility in balancing smoothness and sparsity, leading to improved image fidelity across a wide range of acquisition conditions.

We conducted comprehensive experiments using both numerical phantoms with known ground truths and clinical brain SPECT data to evaluate the effectiveness of the proposed method. The reconstructed images were assessed using both qualitative comparison and quantitative metrics, including the peak signal-to-noise ratio (PSNR) [29,30] and multi-scale structural similarity index measure (MS-SSIM) [31,32]. The proposed method, which introduces a dynamic adjustment mechanism to the ElasticNet regularization, was compared with the standard MLEM algorithm, as well as MLEM with the L1 and L2 regularization and non-dynamic ElasticNet regularization. The experimental results demonstrate that the proposed dynamically regularized ElasticNet MLEM method achieves superior performance in noise suppression, edge preservation, and overall image quality. These advantages suggest that our method holds strong potential for clinical nuclear medicine applications, in which both accuracy and robustness are crucial.

To the best of our knowledge, this is the first study to introduce a *dynamic ElasticNet* strategy within the MLEM framework that is specifically tailored for emission tomography. Furthermore, this study is the first to demonstrate the utility of this approach in clinical brain SPECT imaging, thereby validating its effectiveness under realistic noise conditions and acquisition constraints.

## 2. Definitions and Notations

The fundamental problem of image reconstruction can be formulated as a linear system, in which the goal is to estimate the unknown image $x\in R^{J}$ from the projection data $p\in R^{I}$ measured by the detector as follows:

(1) p=Hx+σ,

where $H\in R^{I\times J}$ represents the system matrix (or projection operator) and $\sigma \in R^{I}$ denotes measurement noise. R denotes the set of real numbers. *J* denotes the total number of pixels in the image, indexed by j=1,…,J, and *I* represents the total number of detector bins in the projection data, indexed by i=1,…,I. Thus, the reconstruction task constitutes an inverse problem, in which the underlying cause *x* must be estimated from the observed data *p*. In tomographic imaging systems, the matrix *H* is determined by the geometric configuration of the detectors and the spatial discretization of the image domain.

In practice, this inverse problem is often ill-posed owing to factors such as measurement noise, limited-angle acquisition, and low-count data, which are conditions that are frequently encountered in nuclear medicine. Even small errors in the projection data can lead to large variations in the reconstructed image. As a result, the direct inversion of *H* is generally unstable or infeasible. Instead, iterative reconstruction methods that incorporate statistical modeling and prior knowledge are widely used to obtain reliable image estimates.

## 3. Maximum-Likelihood Expectation Maximization Algorithm

The MLEM algorithm is a widely used image reconstruction method in nuclear medicine. This algorithm is derived under the assumption that the numbers of both emitted and detected photon decays follow a Poisson distribution. Let $x_{j}$ denote the expected number of disintegrations occurring in pixel *j*, and let $h_{ij}$ represent the element of the system matrix *H* that corresponds to the probability that a photon emitted from pixel *j* is detected by bin *i*. Therefore, the expected number of photons emitted from pixel *j* and detected by bin *i* is expressed as $h_{ij}x_{j}$. Accordingly, the expected number of photons detected by bin *i*, denoted as $p_{i}$, is the sum of contributions from all pixels and can be expressed as follows:

(2) $$p_{i}=\sum_{j=1}^{J}h_{ij}x_{j}.$$

Because the number of photons detected in bin *i* originating from all pixels follows a Poisson distribution, the probability of observing $p_{i}$ photons in bin *i* is expressed as

(3) $$P\left(p_{i}\right)=\frac{\exp\left(-p_{i}\right)\;p_{i}^{p_{i}}}{p_{i}!},$$

where ! denotes the factorial operator. Assuming that the detections in each bin are independent, the joint probability (likelihood) of observing the entire projection data $p=p_{1},p_{2},\ldots ,p_{I}$ given *x* is the product of the individual probabilities:

(4) $$\begin{array}{cc} L(x) & =P(p|x)\\ \; & =P\left(p_{1}\right)\cdot P\left(p_{2}\right)\cdots P\left(p_{I}\right)\\ \; & =\prod_{i=1}^{I}\frac{\exp\left(-p_{i}\right)\;p_{i}^{p_{i}}}{p_{i}!}. \end{array}$$

Taking the logarithm of the likelihood function in Equation (4), we obtain

(5) $$\log\left(L\left(x\right)\right)=\sum_{i=1}^{I}\left(-p_{i}+p_{i}\log\left(p_{i}\right)-\log\left(p_{i}!\right)\right).$$

The log-likelihood function plays a central role in deriving the MLEM algorithm. To determine the most probable estimate of the image *x* that could have generated the observed projection data *p*, we seek the vector x that maximizes log(L(x)).

By setting the partial derivative of the log-likelihood with respect to $x_{j}$ to zero, we obtain the following iterative update scheme of the MLEM algorithm:

$$z_{j}\left(k+1\right)=z_{j}\left(k\right)\cdot f_{j}\left(z\left(k\right)\right),\;z\left(0\right)=x^{0}\in R,$$

where the update function $f_{j}\left(x\right)$ is defined as

(6) $$f_{j}\left(x\right):=\frac{1}{\sum_{i=1}^{I}h_{ij}}\sum_{i=1}^{I}h_{ij}\frac{p_{i}}{\sum_{j^{\prime }=1}^{J}h_{ij^{\prime }}x_{j^{\prime }}}.$$

This update scheme is applied for each j=1,2,…,J over iterations k=0,1,2,…,K−1.

## 4. Proposed System

In this section, we propose an extension of the MLEM algorithm by incorporating regularization techniques. Specifically, we introduce the ElasticNet regularization, which combines the benefits of both the L1 and L2 norms. Furthermore, we propose a dynamic variant that adaptively adjusts the regularization strength during the iterative process. This approach aims to improve the quality of tomographic image reconstruction, particularly under conditions of limited or noisy projection data. Regularization techniques are widely utilized in machine learning, image processing, and statistics to enhance the stability and robustness of the solutions. These techniques are typically applied by reformulating an optimization problem such as the following:

(7) $$\underset{x}{\mathrm{argmin}}||p-{Hx||}_{2}^{2}$$

by adding a regularization term R(x) to penalize undesirable characteristics of the solution:

(8) $$\underset{x}{\mathrm{argmin}}||p-{Hx||}_{2}^{2}-\gamma R\left(x\right),$$

where |·| denotes a norm and γ≥0 is a regularization parameter that controls the strength of the penalty. Among the various regularization methods, the L1 and L2 norms are the most commonly used. L1 regularization encourages sparsity in the solution, whereas L2 regularization promotes smoothness and reduces sensitivity to noise. To leverage the advantages of both, we implement ElasticNet regularization, which is a convex combination of the L1 and L2 terms:

(9) $$R\left(x\right)={\alpha |x|}_{1}+\lambda \left(1-\alpha \right){\left|x\right|}_{2}^{2},$$

where 0≤α≤1 and λ>0 are hyperparameters. When α=1.0, the regularization reduces to L1; when α=0, it corresponds to L2. By adjusting α, one can balance sparsity and smoothness according to the characteristics of the data.

### Dynamic ElasticNet-Regularized EM Reconstruction

We extended the MLEM algorithm by incorporating the ElasticNet regularization term defined in Equation (9).

The resulting update scheme becomes

$$z_{j}\left(k+1\right)=z_{j}\left(k\right)\left(g_{j}\left(z\left(k\right)\right)\right),\;z\left(0\right)=x^{0}\in R$$

where

(10) $$g_{j}\left(x\right):=\frac{1}{\sum_{i=1}^{I}h_{ij}-\gamma R\left(x\right)}\sum_{i=1}^{I}h_{ij}\frac{p_{i}}{\sum_{j^{\prime }=1}^{J}h_{ij^{\prime }}x_{j^{\prime }}}.$$

In this formulation, the regularization term R(x) is introduced with a negative sign in the denominator. This design ensures that the regularization acts as a suppressive factor, effectively dampening the update magnitude in regions in which strong regularization (e.g., high sparsity or smoothness) is desired. If the term were added instead of subtracted, larger regularization values would paradoxically accelerate the update, which would contradict the goal of stabilization and noise suppression. By subtracting γR(x), the method imposes stronger control on updates in which the prior knowledge suggests constraint, thereby enhancing the convergence stability.

This enhanced iterative scheme is expected to yield superior reconstruction quality compared with the conventional MLEM algorithm, especially under conditions with limited or noisy projection data. A key challenge, however, lies in selecting an appropriate value for α to achieve optimal regularization. As opposed to fixing α in advance, we propose a dynamic strategy in which α is updated adaptively at each iteration. This adaptive mechanism enables flexible control over the trade-off between sparsity and smoothness, thereby further enhancing the reconstruction performance. To this end, we define a dynamic update rule for α as a function of the iteration index *k*:

(11) $$\alpha \left(k\right):=\alpha_{0}\cdot \frac{1+\exp(-\omega k)}{2},$$

where $\alpha_{0}>0$ is the initial regularization balance and ω>0 is a decay parameter controlling the rate of change. At iteration zero (k=0), $\alpha \left(0\right)=\alpha_{0}$, allowing the algorithm to initially emphasize sparsity (or smoothness depending on $\alpha_{0}$). As iterations proceed, α(k) smoothly decreases and converges toward $\alpha_{0}/2$, balancing the influence of the L1 and L2 terms adaptively. Formally,

$$\lim_{k\to \infty }\alpha \left(k\right)=\frac{\alpha_{0}}{2},\;\forall \omega >0,$$

and as ω→∞, α(k) rapidly approaches this limit for any fixed *k*.

**Figure 1:**
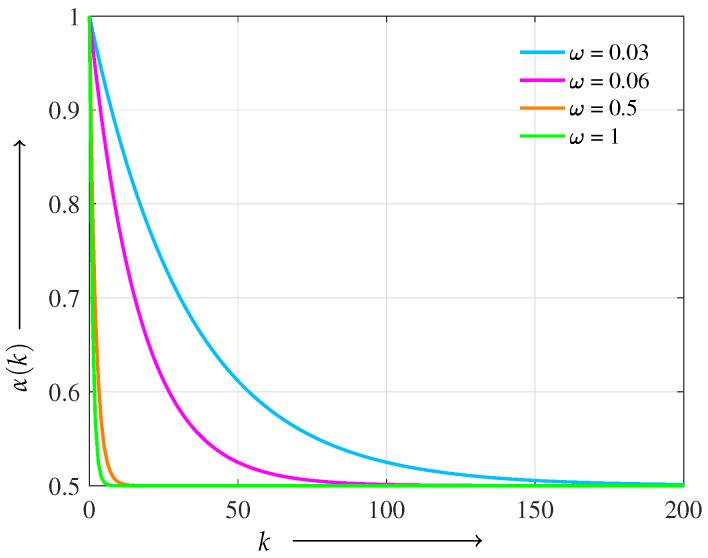
Dynamic scheduling of α(k) for different ω values ($\alpha_{0}=1.0$). The vertical axis shows α(k), and the horizontal axis shows the number of iterations *k*.

Figure 1 shows the behavior of α(k) under different decay parameters ω. We adopt a logistic-type scheduling function owing to its smooth, monotonic convergence and bounded asymptotes, which ensure a gradual and stable transition in regularization balance. Compared with linear or exponential decay, the logistic form avoids abrupt changes or rapid decay, making it particularly suitable for maintaining reconstruction stability throughout the iterations.

To ensure nonnegativity and numerical stability in the update rule (10), the parameters $\alpha_{0}$, ω, and γ must satisfy the condition $\sum_{i}h_{ij}-\gamma R\left(x\right)>0$ for all *j* throughout the iterations. Since R(x) is a convex combination of ${\left|x\right|}_{1}$ and ${\left|x\right|}_{2}^{2}$ modulated by α(k), its maximum impact is bounded by $\alpha_{0}$, and the decay profile is governed by ω. Therefore, choosing sufficiently small γ relative to $\alpha_{0}$ and the dynamic range of R(x) ensures the stability of the denominator and preserves the nonnegativity of the update.

From a computational cost perspective, incorporating the ElasticNet regularization theoretically increases the per-iteration computational complexity from O(IJ) to O(IJ+J), where *I* denotes the number of projection measurements and *J* represents the number of image voxels. However, in practical tomographic settings, the additional O(J) cost introduced by the ElasticNet term is relatively small compared with the dominant forward and backward projection cost of O(IJ). For instance, in a typical setup with a 128×128 image grid (J=16,384) and 90 projection angles with 128 bins each (I=11,520), it is evident that O(IJ)≫O(J), indicating that the projection operations far outweigh the regularization computations. Therefore, the total computational complexity can be effectively approximated as O(IJ+J)≈O(IJ), suggesting that the proposed method introduces only minimal computational overhead compared with standard MLEM.

This dynamic adjustment of α provides several theoretical and practical advantages:

- Improved flexibility: The method adapts to varying noise levels and structural complexity in the image during reconstruction, enabling better noise suppression early on (higher sparsity) and enhanced smoothness preservation later.
- Stable convergence: By modulating the regularization strength throughout the iterations, the algorithm avoids the pitfalls of fixed regularization, which may lead to either over-smoothing or insufficient noise control.
- Balanced regularization: The dynamic trade-off enables maintaining edge sharpness and structural details (favored by L1) while reducing noise and artifacts (favored by L2).
- Enhanced robustness: The approach demonstrates resilience against noise variation and structural heterogeneity, making it particularly effective for clinical imaging scenarios with diverse data quality.

Overall, the proposed dynamic ElasticNet-regularized MLEM algorithm offers a principled and adaptable framework that improves tomographic reconstruction by harmonizing the sparsity and smoothness penalties in a data-driven manner during iterative updates.

## 5. Experiment

This section presents a series of experiments that were conducted to assess the performance of our proposed method. First, we conducted numerical simulations using a modified Shepp–Logan phantom, which is a well-established benchmark for assessing image quality in tomographic reconstruction. Next, we evaluated the effectiveness of our method on anatomically realistic data using a digitized Hoffman 3D brain phantom, which is commonly utilized in nuclear medicine to assess brain imaging algorithms. Finally, we demonstrated the applicability of the proposed method to clinical scenarios by reconstructing clinical images from cerebral blood flow scintigraphy data. All experiments were performed using MATLAB R2025a (MathWorks, Natick, MA, USA) on an M2 Mac mini with 16 GB of memory.

We compared our approach against several baseline methods to validate its effectiveness: the conventional MLEM algorithm and ElasticNet-regularized reconstructions with fixed values of α=1.0 (corresponding to L1 regularization), α=0 (corresponding to L2 regularization), and α=0.5 (representing a fixed balance between the L1 and L2). These were evaluated alongside our proposed dynamic ElasticNet method, in which α was adaptively adjusted during the iterations.

### 5.1. Numerical Example 1 (Shepp–Logan Phantom)

**Figure 2:**
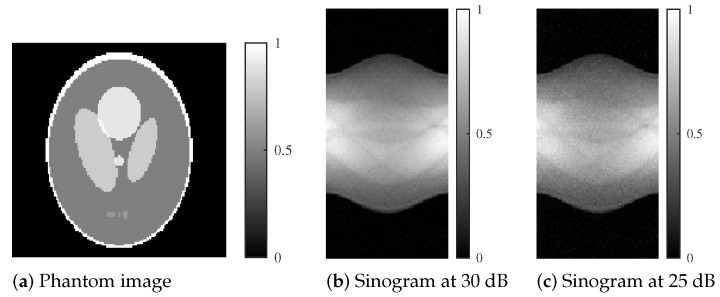
Modified Shepp–Logan phantom and corresponding sinograms at different noise levels.

The modified Shepp–Logan phantom used in our numerical experiments is shown in Figure 2a. The phantom consists of 128 × 128 pixels with intensity values ranging from 0 to 1. Projection data *p* were simulated over 180 degrees using 90 uniformly spaced projection angles. Poisson noise was added to the projections to achieve signal-to-noise ratios (SNRs) of 30 dB and 25 dB.

To assess the performance of our proposed method, we compared it with the conventional MLEM algorithm and the ElasticNet-regularized MLEM with fixed values of α: α=1.0 (L1 regularization), α=0 (L2 regularization), and α=0.5 (balanced ElasticNet). Our proposed dynamic ElasticNet method, where α is adaptively updated during iterations, was also evaluated.

The initial value for each reconstruction was defined as

(12) $$x_{j}^{0}=\sum_{i=1}^{I}p_{i}/\sum_{i=1}^{I}\sum_{j=1}^{J}H_{ij},$$

All methods were iterated for K=200 steps to ensure sufficient convergence. The comparison was conducted using both noise levels: SNR 30 and 25 dB.

We defined the following evaluation function to measure reconstruction error, where $x^{*}$ denotes the ground truth image:

(13) $$E\left(z\right):={\left(\sum_{j=1}^{J}{\left(x_{j}^{*}-z_{j}\right)}^{2}\right)}^{\frac{1}{2}}.$$

A smaller value of E(z) indicates higher similarity between the reconstructed image and the ground truth. In addition, the PSNR and MS-SSIM were employed to quantify the image quality further.

The PSNR was computed as

(14) $$\mathrm{PSNR}=10\cdot \log_{10}\left(\frac{{\mathrm{peak}}^{2}\cdot J}{E{\left(z\right)}^{2}}\right)$$

where peak represents the maximum pixel value. Although the PSNR is easy to implement, it may not always align well with human visual perception [33]. Therefore, the MS-SSIM was used as follows:

(15) $$\mathrm{MS-SSIM}\left(x^{*},z\right)={\left[l_{M}\left(x^{*},z\right)\right]}^{\alpha_{M}}\cdot \prod_{m=1}^{M}{\left[c_{m}\left(x^{*},z\right)\right]}^{\beta_{m}}\cdot {\left[s_{m}\left(x^{*},z\right)\right]}^{\gamma_{m}}.$$

The MS-SSIM is an extension of the SSIM that divides an image into multiple scales (resolutions), calculates the SSIM for each scale, and integrates them into a weighted form. The MS-SSIM is considered to be a more human visual evaluation metric as it not only focuses on image detail but also considers the overall structure and general impression. The parameters in Equation (15) were set to the same values as those in Ref. [31].

### 5.2. Numerical Example 2 (Digitized Hoffman Phantom)

**Figure 3:**
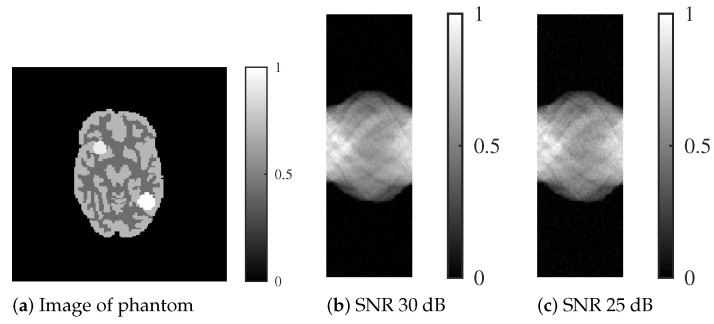
Digitized Hoffman phantom with signal values that simulate accumulation, with sinograms corresponding to each noise level.

We conducted experiments using more practical data to further validate the effectiveness of our proposed method. Specifically, we utilized the phantom shown in Figure 3a, which is a digitized version of the Hoffman 3D brain phantom (Acrobio Co., Ltd. Tokyo, Japan) utilized in nuclear medicine.

The brain parenchyma in the phantom was created with signal values of 0.3 and 0.5 from an arbitrary slice of the Hoffman 3D brain phantom. Furthermore, simulated accumulations of radioactive isotopes were placed at signal values of 0.65 (upper right of the head) and 0.7 (lower left of the head). The Hoffman phantom comprised 128 × 128 pixels, and similar to the Shepp–Logan phantom, SNRs of 30 and 25 dB noise were added to the projection. The projection *p* was simulated with a sampling of 180 degrees and 60 projection directions.

### 5.3. Clinical Example

**Figure 4:**
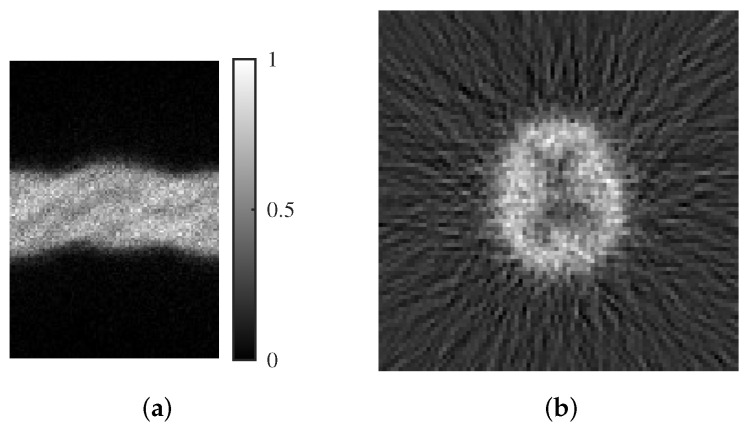
(**a**) Sinogram image and (**b**) image reconstructed by FBP in clinical data.

We employed cerebral blood flow scintigraphy with ^123^I-IMP for the actual clinical data. Projection images were acquired using a Symbia Intevo (Siemens Healthcare Co., Ltd. Forchheim, Germany) SPECT system with an acquisition angle of 360 degrees and 60 projections. The collimator was LEGP, with the reconstructed image comprising 128 × 128 pixels. Attenuation correction was not employed to evaluate the reconstruction quality. A sinogram of an example clinical image and an image reconstructed by FBP are shown in Figure 4. This retrospective study was approved by the Ethics Committee of Tokushima University Hospital (Committee Approval No. 4295-3).

## 6. Results

This section presents the results of the numerical phantom experiments and quantitative evaluations. To assess the performance of our proposed method, we compared it with the conventional MLEM algorithm and ElasticNet-regularized MLEM with fixed values of α: α=1.0 (L1 regularization), α=0 (L2 regularization), and α=0.5 (balanced ElasticNet). Our proposed dynamic ElasticNet method, where α is adaptively updated during iterations, was also evaluated. In addition, we demonstrate the applicability of the proposed method to clinical SPECT brain imaging data.

### 6.1. Numerical Example 1 (Shepp–Logan Phantom)

**Figure 5:**
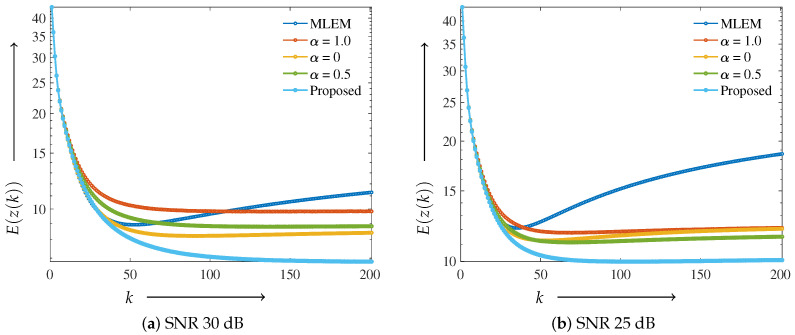
Graph of the evaluation function *E*(*z*(*k*)) and number of iterations *k* using the modified Shepp–Logan phantom: (**a**) shows SNR: 30 dB and (**b**) SNR: 25 dB. A low evaluation function value indicates that the value is close to the true value.

First, we present the results of the modified Shepp–Logan phantom with noise levels of 30 and 25 dB. The graphs of the evaluation function E(z) and the number of iterations *k* are shown in Figure 5a,b. The parameters utilized in Equations (9) and (11) were $\alpha_{0}=0.9$, λ=1.0 and $\alpha_{0}=0.9$, λ=1.1 for 30 and 25 dB, respectively. The value of ω was 0.06 for both noise levels, which was determined as the optimal value from the experimental results. The γ value was set to 0.01 for all regularization methods.

The value of the evaluation function E(z(k)) consistently decreased for all methods in the early stages with a small number of iterations *k*. However, in both the 30 and 25 dB cases, the MLEM method exhibited an increase in E(z(k)) as the number of iterations increased due to noise, failing to achieve stable convergence. The ElasticNet-regularized MLEM with a fixed α=0.5 showed better performance near the final iteration (K=200) in high-noise conditions (25 dB) compared with L1 (α=1.0) and L2 (α=0) regularization. Nonetheless, the proposed method outperformed all other approaches across all noise levels and iteration counts, demonstrating superior stability and convergence behavior.

**Figure 6:**
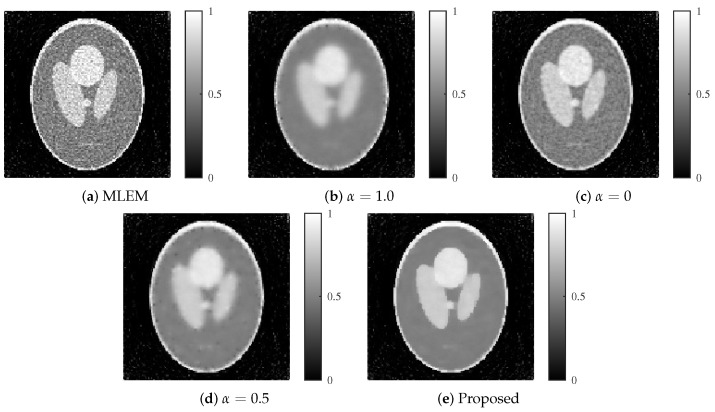
Reconstructed images for each method with an SNR of 30 dB. (**a**) MLEM, (**b**) MLEM with L1 regularization (α=1.0), (**c**) MLEM with L2 regularization (α=0), (**d**) MLEM with ElasticNet regularization (α=0.5 fixed), and (**e**) proposed dynamic ElasticNet regularization. The proposed method exceled in both noise reduction and image detail preservation.

**Figure 7:**
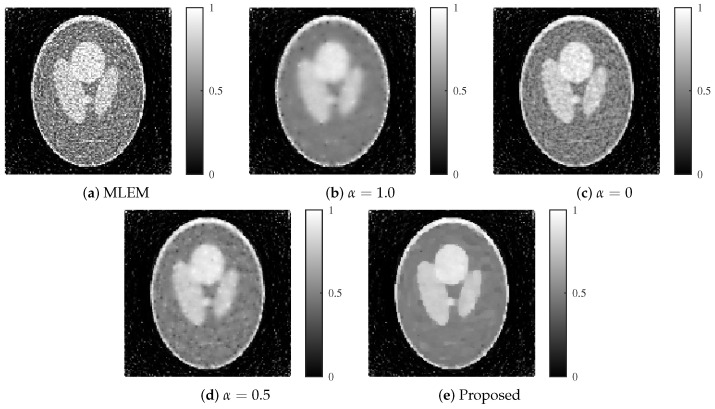
Reconstructed images for each method with an SNR of 25 dB. (**a**) MLEM, (**b**) MLEM with L1 regularization (α=1.0), (**c**) MLEM with L2 regularization (α=0), (**d**) MLEM with ElasticNet regularization (α=0.5 fixed), and (**e**) proposed dynamic ElasticNet regularization. The proposed method exceled in both noise reduction and image detail preservation, even under high noise conditions.

The images reconstructed by each method are presented in Figure 6 and Figure 7. At an SNR of 30 dB, the method with a fixed α=1.0 (L1 regularization) achieved strong noise suppression; however, it also resulted in excessive smoothing, particularly at the edges. In contrast, the method with α=0 (L2 regularization) showed slight noise at 30 dB but exhibited substantial noise artifacts under the more challenging 25 dB condition. The fixed ElasticNet method with α=0.5 provided a balance between noise suppression and edge preservation, yielding intermediate image quality between the α=1.0 and α=0 cases. However, it failed to remove noise sufficiently at 25 dB. In comparison, the proposed method successfully achieved both high-level noise suppression and edge preservation for both noise levels.

We measured the quantitative indices for the reconstructed image for visual evaluation and to explain the results in Table 1.

Table 1 presents the results of the quantitative evaluation using the PSNR and MS-SSIM metrics under SNR conditions of 30 and 25 dB. In terms of the PSNR, the proposed method achieved the highest values among all methods, with 25.459 at 30 dB and 22.084 at 25 dB. The fixed ElasticNet method with α=0.5 also showed competitive performance, particularly outperforming both the α=1.0 and α=0 settings at 25 dB. Nevertheless, its performance remained lower than that of the proposed method.

Similarly, for the MS-SSIM metric, the proposed method attained the best scores, achieving 0.928 at 30 dB and 0.869 at 25 dB, indicating superior structural similarity and visual quality. While the fixed ElasticNet (α=0.5) again showed balanced performance between noise suppression and structural preservation, it did not exceed the proposed method. These results confirm the effectiveness of the proposed dynamic ElasticNet approach under both noise conditions.

### 6.2. Numerical Example 2 (Digitized Hoffman Phantom)

**Figure 8:**
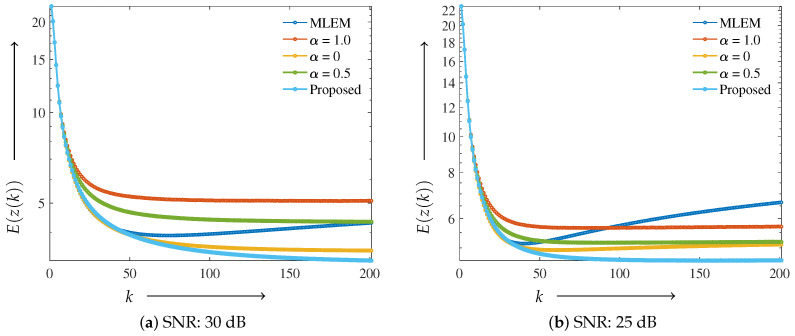
Graph of the evaluation function E(z(k)) and number of iterations *k* using the digitized Hoffman phantom: (**a**) SNR: 30 dB and (**b**) SNR: 25 dB. A low evaluation function value indicates that the value is close to the true value.

The performance of the proposed method was evaluated using more clinically relevant images. Similar to the Shepp–Logan phantom, a plot of the evaluation function is shown in Figure 8. The parameters utilized in Equations (9) and (11) were $\alpha_{0}=0.2$, λ=0.1 and $\alpha_{0}=0.4$, λ=0.1 for 30 and 25 dB, respectively.

Similar to the Shepp–Logan results, the MLEM method diverged as the number of iterations increased at SNR levels of both 30 and 25 dB. For the fixed ElasticNet method with α=0.5, intermediate performance was observed between α=1.0 and α=0 at 30 dB. However, at 25 dB, the α=0 case resulted in a lower evaluation function value than that for α=0.5. In contrast, the proposed method consistently exhibited stable performance across all noise levels and maintained low evaluation function values, even under the high-noise condition of 25 dB.

**Figure 9:**
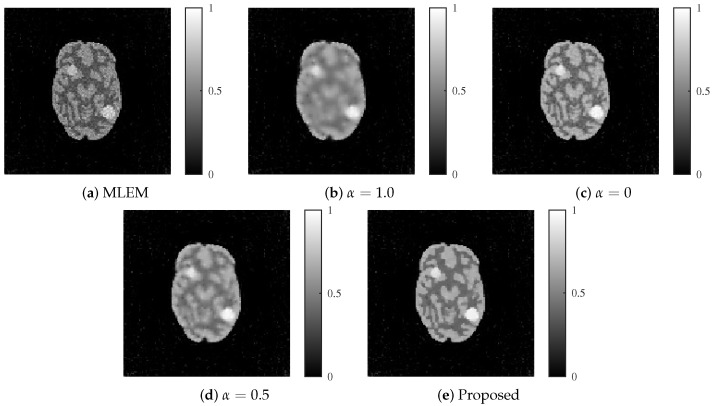
Reconstructed images for each method with an SNR of 30 dB. (**a**) MLEM, (**b**) MLEM with L1 regularization (α=1.0), (**c**) MLEM with L2 regularization (α=0), (**d**) MLEM with ElasticNet regularization (α=0.5 fixed), and (**e**) proposed dynamic ElasticNet regularization. The proposed method exceled in both noise reduction and image detail preservation.

**Figure 10:**
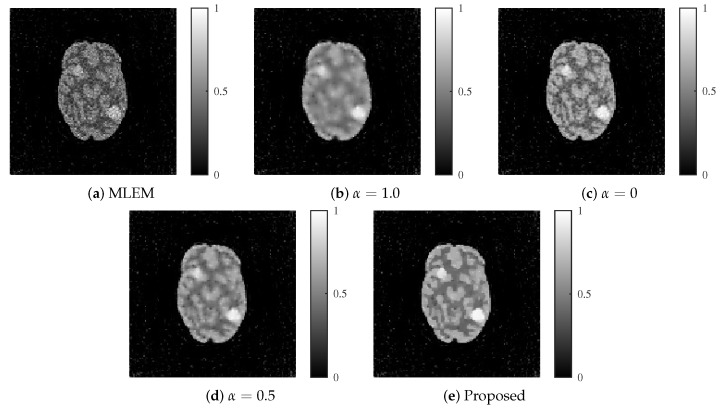
Reconstructed images for each method with an SNR of 25 dB. (**a**) MLEM, (**b**) MLEM with L1 regularization (α=1.0), (**c**) MLEM with L2 regularization (α=0), (**d**) MLEM with ElasticNet regularization (α=0.5 fixed), and (**e**) proposed dynamic ElasticNet regularization. The proposed method excels in both noise reduction and image detail preservation, even under high noise conditions.

The reconstructed images produced by each method are shown in Figure 9 and Figure 10. The proposed method achieved an optimal balance between noise and resolution compared with the other methods, even at noise levels of 30 and 25 dB.

The measured quantitative indices are listed in Table 2.

The quantitative evaluation results for the Hoffman phantom are summarized in Table 2. At an SNR of 30 dB, the proposed method achieved the highest PSNR (31.982) and MS-SSIM (0.972), outperforming all other methods. α=0 also exhibited relatively high performance with a PSNR of 31.317 and an MS-SSIM of 0.970, whereas the fixed ElasticNet method (α=0.5) showed intermediate results between the L1 and L2.

At an SNR of 25 dB, the proposed method maintained its superiority, achieving the highest PSNR (28.839) and MS-SSIM (0.932), thereby indicating its robustness under higher noise conditions. Although L2 again performed well (PSNR: 27.980 and MS-SSIM: 0.928), the proposed method consistently outperformed it. In contrast, the L1 method yielded the lowest PSNR and MS-SSIM values at both noise levels, suggesting over-smoothing and insufficient structural preservation.

These results highlight that the proposed adaptive ElasticNet approach effectively balances noise suppression and edge preservation, leading to superior quantitative image quality across different noise conditions.

### 6.3. Clinical Data

**Figure 11:**
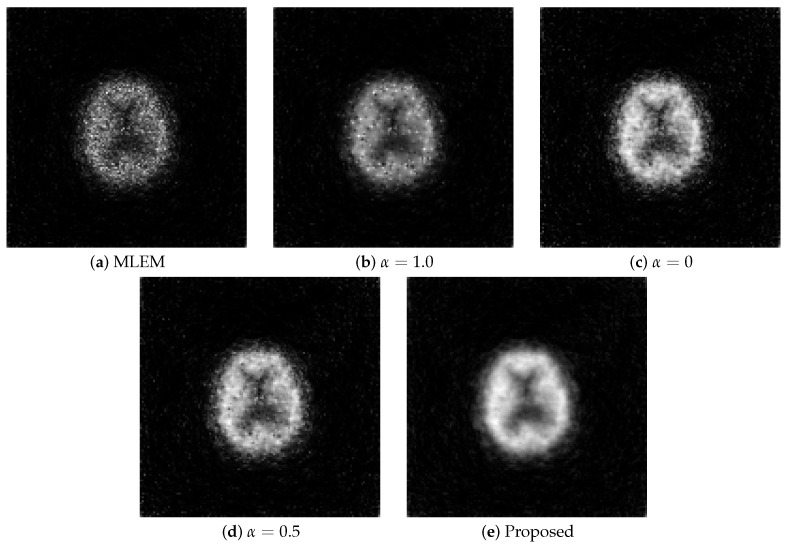
Reconstructed images for each method using clinical data: (**a**) MLEM, (**b**) MLEM with L1 regularization (α=1.0), (**c**) MLEM with L2 regularization (α=0), (**d**) MLEM with ElasticNet regularization (α=0.5 fixed), and (**e**) proposed dynamic ElasticNet regularization. The proposed method also achieved superior performance in clinical images.

The results obtained from the clinical data are shown in Figure 11. The parameters utilized in Equations (9) and (11) were $\alpha_{0}=0.01$ and λ=3.5.

The MLEM method generated noisy images compared with the other methods, rendering it unsuitable for observing images. Although the regularization with the L1 and L2 aided in noise reduction, the images contained more noise than those generated using the proposed method, failing to provide adequate noise suppression. Similar to the numerical phantom experiment, the proposed method outperformed the others the most in terms of noise and resolution.

**Figure 12:**
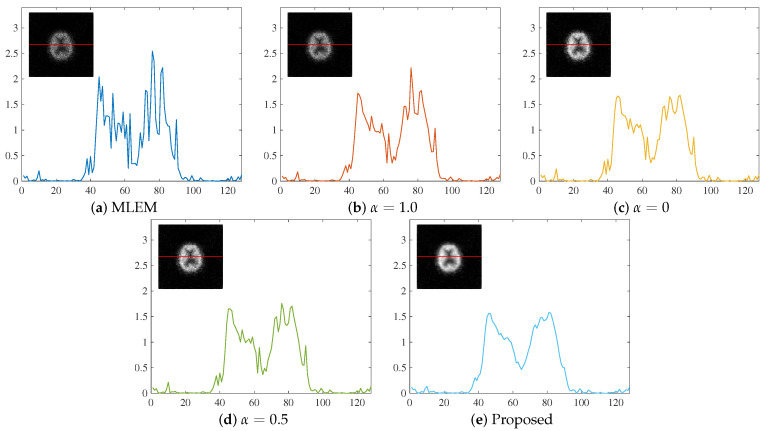
Density profiles of (**a**) MLEM, (**b**) α=1.0, (**c**) α=0, (**d**) α=0.5, and (**e**) the proposed method for reconstructed images along the horizontal line. The position of the acquired density profile is represented by a red line in the density profile plot.

To objectively evaluate the reconstructed images, the density profile of the central parts is shown in Figure 12.

The values on the vertical axis are normalized for ease of comparison. Similar to the visual evaluation, the proposed method excelled in noise suppression compared with the other methods. Although MLEM-L2 also exhibited strong noise suppression capabilities, the proposed method proved to be more effective in suppressing image noise.

## 7. Discussion

This study proposed a novel MLEM algorithm incorporating dynamic ElasticNet regularization for image reconstruction in nuclear medicine imaging and assessed its effectiveness. The proposed method is based on ElasticNet regularization, which combines the advantages of the L1 and L2 norms. In addition, an adaptive weighting mechanism is introduced that dynamically adjusts the balance between the L1 and L2 regularization terms during the iterative reconstruction process according to the image and projection data characteristics.

Experimental results using numerical phantom data and actual clinical SPECT brain images demonstrated that our proposed method consistently outperformed the conventional MLEM algorithm and MLEM with fixed ElasticNet regularization parameters (α=1.0 for L1, α=0 for L2, and α=0.5 for fixed ElasticNet) in terms of noise suppression, edge preservation, and overall image quality.

In numerical experiments with the modified Shepp–Logan phantom and the more structurally complex digitized Hoffman phantom under noise levels of SNRs 30 and 25 dB, the proposed method showed stable convergence in all cases. The quantitative evaluations summarized in Table 1 and Table 2 confirmed the superior performance of the proposed method across all PSNR and MS-SSIM metrics, particularly demonstrating strong noise robustness at the lower SNR of 25 dB.

The adaptive weighting mechanism enabled more effective balancing of noise suppression and edge preservation throughout the iterative process compared with fixed-α methods. Early iterations emphasized noise reduction by stronger regularization, whereas later iterations preserved the edge details by dynamically adjusting the regularization balance. This adaptivity contributed significantly to the consistent high-quality reconstruction of the method across different noise levels and phantom complexities.

Clinical SPECT brain image evaluations further validated that the proposed method effectively suppressed noise while accurately depicting clinically important fine structures, highlighting its potential for practical clinical use.

The selection of regularization parameters in the proposed method was based on empirical tuning rather than systematic optimization. In our experiments, we found that it is essential to adjust the parameters according to the imaging conditions, such as the structure of the target object, the number of projection angles, and the noise level. These factors significantly influence both the convergence behavior and the quality of the reconstructed images. Notably, among the parameters used in the dynamic ElasticNet regularized EM algorithm, the regularization strength parameter γ=0.01 and the decay parameter ω=0.06, which controls the dynamic update of α(k), were kept constant across all experiments. These values were selected to ensure the numerical stability and nonnegativity of the update factors, which are critical for maintaining the multiplicative structure of the EM framework. In contrast, effective reconstruction performance was achieved by tuning only two parameters: the initial L1/L2 balance $\alpha_{0}$ and the weighting coefficient λ of the L2 term. This observation suggests that the proposed method is relatively robust and that minimal parameter adjustments can yield satisfactory results under various imaging conditions. Nevertheless, further exploration of automated or data-driven parameter selection strategies remains an important direction.

However, this study has some limitations. The performance of the method depends on appropriate hyperparameter selection for adaptive weighting, and improper tuning may lead to suboptimal results or slower convergence. Moreover, due to the lack of ground truth in clinical data, no-reference metrics or radiologist-based evaluations were not performed in this study, which remains a limitation to be addressed in future work.

Although this study focused on nuclear medicine, the proposed framework can be extended to other imaging modalities, such as CT, by appropriately adapting the system matrix *H* and the forward model. This highlights the potential of the method as a generalizable inverse problem solver in medical image reconstruction across various modalities.

Overall, the proposed MLEM algorithm with dynamic ElasticNet regularization demonstrated stable and superior performance compared with conventional fixed-parameter methods, confirming its potential to provide higher-quality images in modern clinical nuclear medicine, in which low-dose and short-time acquisitions are increasingly common. To the best of our knowledge, this is the first work to incorporate a dynamic ElasticNet strategy into MLEM for emission tomography and to validate it on clinical brain SPECT data.

## 8. Conclusions

We have proposed a dynamic ElasticNet-regularized MLEM algorithm that adaptively balances the L1 and L2 regularization to enhance image reconstruction in nuclear medicine. Experiments with numerical phantoms and clinical SPECT images demonstrated that the proposed method consistently outperformed conventional MLEM and fixed ElasticNet approaches in noise suppression and edge preservation, particularly under low-SNR conditions. The adaptive weighting mechanism improved the flexibility across different noise levels, contributing to stable convergence and superior image quality, supporting its potential for clinical applications in low-dose and fast imaging. To the best of our knowledge, this is the first study to incorporate a dynamically adjusted ElasticNet strategy within the MLEM framework for emission tomography.

## Figures and Tables

**Table 1:** Results of quantitative indicators.

	Method	SNR 30 dB	SNR 25 dB
PSNR	MLEM	21.094	16.763
	α=1.0	22.290	20.469
	α=0	23.642	20.516
	α=0.5	23.228	20.913
	Proposed	25.459	22.084
MS-SSIM	MLEM	0.873	0.783
	α=1.0	0.904	0.851
	α=0	0.897	0.820
	α=0.5	0.911	0.841
	Proposed	0.928	0.869

**Table 2:** Results of the quantitative indicators.

	Method	SNR 30 dB	SNR 25 dB
PSNR	MLEM	29.478	25.694
	α=1.0	28.014	27.010
	α=0	31.317	27.980
	α=0.5	29.405	27.838
	Proposed	31.982	28.839
MS-SSIM	MLEM	0.968	0.923
	α=1.0	0.954	0.920
	α=0	0.970	0.928
	α=0.5	0.964	0.927
	Proposed	0.972	0.932

## Data Availability

All data used to support the findings of this study are included within the article.
